# Personalized Sleep Hygiene and Structured Rest Interventions Improve Sleep Quality and Energy in a Rotating Shift Worker: A Case Report

**DOI:** 10.7759/cureus.76497

**Published:** 2024-12-28

**Authors:** Joshua A Jogie

**Affiliations:** 1 Faculty of Medical Sciences, The University of the West Indies, St. Augustine, TTO

**Keywords:** family support, fatigue, occupational health, rotating shifts, sleep hygiene

## Abstract

A 38-year-old paint technician who worked rotating shifts reported mild sleep disruptions and increased fatigue. The company’s medical staff reviewed his work patterns, rest habits, and home environment. They introduced a personalized sleep hygiene program and adjusted his break schedule, allowing short, structured rest periods. They also provided simple handouts on proper home lighting, relaxation methods, and guidance on coordinating family meals. Within a few weeks, his sleep quality improved, and his energy levels stabilized. This case shows that a focused, family-oriented approach can help workers adapt to challenging shift patterns and maintain better health.

## Introduction

Shift work is a widespread practice in many industries and is essential for sectors that must operate around the clock. Workers in manufacturing, healthcare, transportation, and service industries commonly alternate between different shifts to maintain continuous operations. This scheduling approach is often necessary, but it poses well-documented challenges to workers’ sleep and overall health. Disruptions to normal circadian rhythms can impair an individual’s ability to achieve restful sleep, and these disruptions can accumulate over time and affect cognitive function, mood, and productivity [1,2]. Over the past few decades, research has demonstrated a clear link between rotating shift work and disturbances in sleep patterns, with workers often reporting poor sleep quality, daytime fatigue, and difficulties with alertness and concentration [3]. Such problems can have serious implications for both employee well-being and workplace safety, as fatigued workers are at higher risk for accidents and injuries [4]. Studies have identified sleep disturbances in shift workers as a significant concern, and the importance of addressing this issue has grown as more industries continue to rely on nonstandard schedules.

Many biological factors underlie the difficulties that shift workers face. The human body operates on an internal circadian clock, which aligns many physiological processes with the external light-dark cycle. When individuals work at night or rotate through different shifts, their exposure to natural light patterns changes, and their usual sleep times often shift as well. This misalignment can lead to chronic sleep loss and circadian rhythm disorders that erode overall health [5]. Over time, shift workers may develop patterns of insufficient or fragmented sleep. Fatigue can worsen, and the long-term consequences may include not only reduced work performance but also metabolic and cardiovascular problems. These concerns have led researchers and occupational health professionals to explore targeted interventions that help workers maintain healthier sleep habits even when facing irregular schedules.

Efforts to improve sleep quality among shift workers often revolve around several core strategies. One approach is to optimize the sleep environment, such as advising on proper lighting, reducing noise, and controlling bedroom temperature. Another involves setting consistent sleep routines and relaxation techniques that promote more efficient rest, even with irregular work hours. Employers and health professionals may also consider structured rest breaks during shifts, strategic caffeine use, and education on the timing of meals to align better with the worker’s internal clock [6]. Technological supports, such as wearable devices to monitor sleep patterns or light therapy devices to help reset circadian rhythms, may also aid in managing these challenges. However, not all interventions succeed equally for every individual, which highlights the importance of personalized programs.

Research shows that personalized interventions often yield better results than one-size-fits-all solutions. Every worker has a unique lifestyle, family environment, and personal preference for sleep routines. For a worker who must coordinate family activities, help children with homework, or prepare meals at variable times, a rigid standard approach may not be practical. A personalized sleep hygiene program can address the specific needs of an individual, considering their home environment, social commitments, and personal habits. In addition, family involvement may play a crucial role. Support from family members can help align household activities with the worker’s new sleep schedule, making it easier to maintain healthy sleep practices in the long term. Educating not just the worker but also the family on the importance of proper lighting at home, quiet periods for sleep, and meal coordination can create a more supportive environment that strengthens the worker’s ability to adapt to shift work demands [7].

Occupational health teams can play a central part in this process. They have the opportunity to conduct thorough evaluations of workers who report difficulties related to shift schedules. These evaluations can include interviews about sleep routines, assessments of fatigue levels, and inquiries into home conditions that might affect sleep quality. In some cases, simple questionnaires can help identify sleep disturbances. If warranted, more objective measures such as actigraphy or polysomnography can be employed. Actigraphy involves wearing a device that tracks activity and rest patterns over several days, providing an overview of sleep-wake rhythms in a natural setting. While this level of investigation may not always be needed for mild cases, it can guide more complex interventions. Ultimately, the goal is to identify modifiable factors and introduce targeted changes that help the worker achieve a better balance between rest and work demands.

Support from employers is critical in implementing these strategies. Companies that value employee health recognize that workers who sleep well perform better and are less likely to experience accidents. Offering health education sessions, providing written materials about sleep hygiene, and adjusting work schedules to allow for structured rest breaks all contribute to an environment where employees can thrive. In some settings, simple measures such as optimizing shift rotations to reduce abrupt changes in start times can have meaningful effects. Short rest breaks during a long shift can alleviate the buildup of sleepiness, and strategically timed exposure to bright light can help maintain alertness during nighttime work hours [8]. Providing these forms of support can highlight a company’s dedication to its workforce, reflecting a proactive stance in promoting well-being rather than merely responding to health issues as they arise.

In the case of a 38-year-old paint technician, for example, mild sleep disturbances and increased fatigue can be early signals that the body and mind are struggling to adapt to an irregular schedule. Identifying the problem at this stage allows for timely interventions before chronic fatigue sets in and leads to more serious health consequences. The company’s medical staff can initiate a careful evaluation, ask questions about sleep timing, assess the quality of sleep, and identify which aspects of the worker’s daily routine might be contributing to the problem. In many cases, relatively small but well-designed changes can yield significant improvements. Providing guidance on proper lighting at home might mean suggesting ways to darken the bedroom during the day or recommending light therapy in the early evening. Relaxation techniques can help the worker fall asleep more quickly, and family meal coordination can ensure that the worker does not have to juggle meal preparation and family duties during their designated rest times. Encouraging short, structured rest breaks during work shifts can help stabilize energy levels. Within a few weeks, such measures often lead to improvements in sleep quality and a noticeable increase in daytime energy.

This sort of proactive and family-oriented approach can balance occupational demands with personal well-being. It can make a difference not only in how well an individual adjusts to rotating shifts but also in their long-term health and job satisfaction. While occupational demands will not disappear, addressing sleep disturbances early and comprehensively can help workers maintain stable energy levels and reduce feelings of chronic fatigue. It also sets a positive example for other employees, showing that the company invests in practical solutions to common health issues related to shift work. By doing so, employers send a message that acknowledges the importance of sound sleep and circadian health, which may enhance overall morale and reduce absenteeism or turnover.

Several studies have examined how various interventions - ranging from scheduling adjustments to educational materials - can improve sleep in shift workers. Reducing irregular shift rotations, offering flexible break times, and encouraging personal rest strategies have all been associated with better outcomes. While not all interventions will work for everyone, tailoring them to suit an individual’s circumstances can often yield more stable results. This personalization reflects the complexity of human biology and social life, as sleep is not just a biological function but also influenced by home routines, cultural practices, and interpersonal relations. When companies and health professionals work closely with employees and their families, they create a supportive network that helps guide the worker through changes that might otherwise feel daunting. Over time, the worker learns habits that maintain better sleep quality and promote a stable level of alertness, even as schedules continue to rotate.

## Case presentation

A 38-year-old male paint technician presented to the company’s medical staff with mild sleep disturbances and increased daytime fatigue. He worked a rotating shift schedule that varied over the course of several weeks, including day, evening, and night shifts. He stated that he had difficulty maintaining consistent sleep patterns and often felt tired during work hours. He mentioned that he sometimes felt less alert than usual and worried about his ability to maintain productivity and focus. He also expressed mild irritability at home, noting that it was becoming more challenging to help with family activities due to feelings of tiredness.

His medical history included no significant chronic conditions, and he was not taking any prescription medications. He reported no ongoing respiratory, cardiac, or endocrine issues. He did not smoke and occasionally consumed alcohol, though not in large quantities. He had worked in the paint department for several years and had grown accustomed to certain aspects of the job. However, he noted that the rotating shifts had become more difficult in recent months. He said he tried to sleep whenever possible but found it challenging to maintain a regular sleep schedule. Sometimes he would try to stay awake longer on his days off to spend time with his family, which seemed to further disrupt his rest.

To evaluate his condition, the medical staff collected detailed information about his sleep patterns. They asked him to maintain a sleep diary for two weeks. He recorded his bedtimes, wake times, subjective sleep quality, and episodes of waking during the night. This diary showed inconsistent sleep schedules that changed dramatically from one day to the next, correlating with his shift assignments. On days following night shifts, he often slept at irregular times and for shorter durations. On days after evening shifts, he might attempt to sleep longer but often reported difficulty initiating sleep due to residual alertness. On days following day shifts, he tended to sleep more normally but still complained of mild fatigue.

The medical team also administered a standard sleepiness and fatigue questionnaire, such as the Epworth Sleepiness Scale, to quantify his level of daytime sleepiness. He scored in a range indicating mild to moderate daytime sleepiness. They performed a brief physical exam to rule out any obvious physical contributors to his fatigue. Vital signs were stable, and there were no abnormal findings on a basic examination of the respiratory or cardiovascular systems. Given the mild nature of his complaints, more complex investigations such as polysomnography were not considered immediately necessary. Instead, the team focused on evaluating his work schedule, home environment, and daily routines. They noted that his primary complaint was not severe insomnia or pathological sleep disorders, but rather difficulty adjusting to the rotating shift pattern.

They also considered the possibility of underlying conditions like thyroid dysfunction or anemia that could contribute to fatigue. They ordered a routine set of laboratory tests, including a complete blood count, thyroid function tests, and a basic metabolic panel. The results of these tests were within normal limits. This reduced the likelihood that a medical disorder unrelated to shift work was responsible for his symptoms.

During the interview, the patient mentioned that his home environment could be improved for better sleep. His bedroom blinds did not fully block morning light, and his room was near a common living area. Noise from family activities sometimes made it hard for him to rest during the day after a night shift. He said that he had not tried any targeted relaxation techniques and mainly relied on catching up on sleep whenever he had the chance. He did not have a consistent strategy for adjusting to new shift patterns, and he admitted that he often worried about missing out on family time if he chose to rest when others were awake.

The medical staff discussed these findings with the patient and decided to implement a personalized sleep hygiene program. They focused first on small changes that could be made easily. They recommended that he adjust his bedroom lighting. They advised installing heavier curtains or blinds to create a darker environment conducive to rest during the day. They suggested using earplugs or a white noise machine if noise from the rest of the household could not be minimized. Additionally, they encouraged him to set a more consistent schedule for sleep whenever possible, even if it meant going to bed at unusual times to align with his shifts.

To address the rotating shifts, the medical staff reviewed his break schedules. They collaborated with his supervisor to allow short, structured rest periods during his shifts. These breaks did not disrupt the workflow significantly, but they gave him predictable times to rest and recover. He was encouraged to engage in simple relaxation techniques before attempting to sleep, such as controlled breathing or listening to quiet music. He received printed handouts with suggestions for preparing for sleep, including reducing exposure to bright light before bedtime, avoiding caffeine late in the shift, and keeping meal times consistent with the sleep schedule.

The team also explored family involvement. They recognized that the patient’s household played an important role in supporting or undermining his sleep habits. The patient’s partner and children needed to understand that small adjustments to their routines might help him rest better. To facilitate this, the company’s medical staff provided guidance on meal coordination. They suggested that the family consider serving the main meal at a time that fits the patient’s schedule rather than always following traditional meal times. They offered simple instructions on how to ensure that the home environment remains quiet during the hours he must sleep. They shared relaxation techniques that could be performed as a family, such as establishing a calm atmosphere in the home before the patient’s bedtime.

The patient’s shift supervisor agreed to adjust his break schedule so that he had a short rest opportunity during the most challenging part of the shift. This small but structured break allowed him to relax briefly, lowering the level of perceived fatigue. Additionally, the medical staff highlighted the importance of consistency. They asked the patient to keep track of his new sleep routine and to return for follow-up after a few weeks to report any changes. They planned to reassess his sleep quality using the same diaries and sleepiness scale to see if the interventions were helping.

Over the following weeks, the patient began to implement the recommended changes. He installed thicker curtains, which effectively blocked sunlight and helped him sleep better during daytime hours. He tried relaxation breathing exercises before going to bed. His family agreed to coordinate their activities so that the home was quieter during his rest periods. He also took advantage of the structured rest breaks during his shifts, using them as a time to unwind and disconnect briefly from work stress. He paid more attention to when he ate his meals, trying to have a light meal before sleep and a more substantial meal at a stable time that the entire family could plan around.

After about four weeks, he reported noticeable improvements. He felt that he could fall asleep more easily during the day, and he woke feeling more rested. His self-reported sleep quality improved. He recorded fewer awakenings, and his total sleep duration became more stable. On days after night shifts, while still not ideal, he was able to achieve more consistent rest. His daytime fatigue lessened, and he felt more alert during work hours. The follow-up Epworth Sleepiness Scale score dropped closer to a normal range, indicating that he was feeling less daytime sleepiness. He noted that he had more energy to engage in family activities and that his mood had improved as well.

The company’s medical staff observed that these changes required minimal resources. They provided basic educational materials, simple adjustments to the patient’s work schedule, and guidance on improving the home sleep environment. The family’s willingness to coordinate mealtimes and create a quiet atmosphere during his rest periods supported his adaptation to a challenging schedule. While the patient’s rotating shifts could not be eliminated, the combination of personalized sleep hygiene, environmental adjustments, structured rest breaks, and family involvement helped restore a better balance between his occupational demands and personal well-being.

## Discussion

The case presented here shows that a personalized, family-oriented approach can improve sleep quality and reduce fatigue in a worker dealing with rotating shift schedules. The patient’s mild sleep disturbances and increased daytime tiredness reflected challenges common to shift workers who must adapt to nonstandard hours. Evidence suggests that shift work often leads to circadian misalignment, reduced sleep duration, and more fragmented rest. Such disruptions have been linked to impaired cognitive performance, mood disturbances, and lower quality of life. Over time, these issues can contribute to physical health problems, including metabolic changes, cardiovascular risk, and altered immune responses. Thus, addressing even mild symptoms early may prevent more severe complications and improve both occupational safety and personal well-being.

The intervention described in this case relied on practical steps that acknowledged the patient’s specific schedule, home environment, and family responsibilities. The medical staff chose to personalize sleep hygiene advice and adjust shift break times to allow structured rest periods. Studies have shown that simple, targeted measures can help workers cope better with irregular schedules. For example, adjusting sleep timing, controlling exposure to bright light, and ensuring stable rest periods may help workers maintain better alertness and overall functioning. In a broad sense, occupational health professionals can implement these measures without significant cost or disruption to the workflow.

The role of proper lighting stands out. In typical day-oriented schedules, exposure to daylight helps synchronize the body’s circadian clock, promoting wakefulness in the morning and sleepiness at night. But shift workers often sleep during daylight hours and work at night, which flips normal biological cues. Managing light exposure can minimize circadian disruption. Strategies include using blackout curtains to reduce morning light and introducing bright light during certain phases of the shift to maintain alertness. Previous research has shown that timed exposure to bright light can help align the body’s internal clock with nonstandard work times, and well-controlled exposure can improve sleep and reduce fatigue [9]. Similar recommendations have been supported for healthcare workers and other professionals who face irregular schedules.

Break schedules also matter. A short, structured rest period can help mitigate sleep pressure that accumulates during long or late shifts. Studies indicate that planned naps or rest breaks at work may reduce fatigue and enhance subsequent performance. Even brief rest periods can restore some alertness and improve mood. Research has found that scheduled breaks and rest opportunities can enhance employee well-being without negatively affecting productivity [10]. In fact, many organizations now recognize the importance of strategic rest breaks as a means to sustain performance over extended or irregular shifts.

Encouraging family support is another key aspect of this case. Shift work does not affect only the worker; it impacts the entire household. Family members’ activities can disturb sleep if they occur during the worker’s rest times. Providing guidance on meal coordination and quiet periods can help align family routines with the worker’s sleep schedule. In this scenario, simple steps like adjusting family meal times and reducing noise levels during the worker’s main sleep period helped stabilize his sleep patterns. Although scientific literature often focuses on organizational and individual factors, the role of social and family support in helping shift workers cope with irregular schedules is also recognized [11]. When families understand the importance of sound sleep, they can create an environment more conducive to rest, thereby reducing tension and misunderstandings that might arise when one household member’s schedule differs so markedly from others.

Relaxation techniques and stress reduction methods play a part in improving sleep quality. Stress often worsens sleep difficulties, and poor sleep can increase stress, creating a vicious cycle. Providing the worker with handouts on relaxation methods, such as controlled breathing, progressive muscle relaxation, or simple mindfulness exercises, may help reduce pre-sleep arousal. Research supports the use of stress management and relaxation techniques to improve sleep initiation and quality in shift workers [12]. When workers enter their rest period feeling calmer, they tend to fall asleep more easily and stay asleep longer.

It is worth noting that the severity of the patient’s initial complaints was mild. Mild sleep disturbances may respond well to non-pharmacological interventions and lifestyle modifications. More severe cases might require different approaches, including pharmacological treatments. However, promoting good sleep hygiene first is often recommended. Non-pharmacological methods are generally preferred as a first-line approach because they carry fewer risks and can create long-term behavioral changes that persist even if shift patterns fluctuate [13]. In this case, the early intervention helped prevent the escalation of symptoms and allowed the patient to regain stable energy levels within a few weeks.

Another important aspect is the demonstration of the employer’s commitment to the worker’s health. By providing personalized guidance, adjusting schedules, and offering educational materials, the company signaled that it valued the well-being of its employees. This can improve trust and morale in the workforce. Workers who feel supported by their employers are often more motivated and less likely to seek other employment opportunities. This aligns with research showing that supportive workplace policies and practices can enhance job satisfaction and reduce turnover [14]. In the long run, investing in employee health may also reduce absenteeism and healthcare costs associated with chronic fatigue and sleep-related disorders.

The interplay between occupational demands and personal well-being is complex. Shift workers face demands that clash with their natural biological rhythms. Addressing these conflicts is not always straightforward, but small changes can have measurable effects. Tailored interventions, rather than generic advice, acknowledge that each worker’s life circumstances differ. Some workers may live alone, while others may have children or older parents at home. The resources and time available to improve sleep conditions can vary widely. By considering the individual’s situation, health professionals can recommend measures that are realistic and sustainable. Evidence suggests that tailored interventions are more effective in promoting long-term adherence to health guidelines [15]. Workers who feel that interventions were designed specifically for them may be more likely to follow through and maintain the new habits over time.

This case also reminds us that monitoring and follow-up are essential. Evaluating progress through sleep diaries and standardized questionnaires can help confirm whether interventions are working. If improvements are not seen, adjustments can be made. For example, if blackout curtains are not sufficient to block noise, suggesting the use of earplugs or rearranging furniture might help. If short rest breaks at work do not ease fatigue, further schedule refinement could be needed. The willingness to re-evaluate and modify interventions is an important component of a successful occupational health program. Evidence-based guidelines suggest that continuous assessment and iterative improvements can optimize the benefits of interventions for shift workers [16].

This case aligns with previous research on managing shift work-related sleep issues, which stresses a combination of environmental, behavioral, and educational strategies. While not all workplaces may have the resources or the flexibility to implement the exact measures described, many of these steps require minimal investment. Simple educational materials, communication with families, and minor adjustments to break schedules can bring meaningful change. Even workers who have limited control over their shift patterns can improve sleep quality by making small changes at home and using rest breaks strategically.

The case described here focused on a single individual. It should not be assumed that all shift workers will respond similarly. Some may need more intensive interventions, such as scheduled light therapy, professional counseling, or pharmacological assistance. Others may find it harder to achieve family support or face structural barriers at work that prevent schedule adjustments. Further research could compare different intervention strategies and identify which methods work best for particular types of shift workers. For example, certain industries may present unique challenges. Healthcare workers often face unpredictable patient care needs, while manufacturing workers may have less control over their environment. Identifying the most effective interventions for each setting could help develop more comprehensive guidelines.

This case highlights that mild sleep disturbances in a shift worker can respond positively to a personalized, family-oriented approach. Simple interventions based on recognized principles of good sleep hygiene, environmental management, and social support can improve sleep quality and help workers maintain stable energy levels. Though no single solution will fit every scenario, an individualized plan that respects the worker’s unique circumstances can yield meaningful improvements. By acknowledging both work and home environments, occupational health professionals can create strategies that support long-term health and well-being for those who must contend with rotating or irregular shift schedules.

## Conclusions

This case report shows that a personalized sleep hygiene plan can help a shift worker improve sleep quality and reduce fatigue. Adjusting break times, guiding family routines, and using simple relaxation methods can support better rest and more stable energy levels. This case suggests that even small, practical changes can help workers cope with rotating schedules and maintain their well-being.
